# HearCog Trial‐ Findings from FDG‐PET

**DOI:** 10.1002/alz70856_100129

**Published:** 2025-12-25

**Authors:** Heidi Espedal, Dona M P Jayakody, Adriano Polpo, Ford H Andrew, Leon Flicker, Osvaldo P Almeida, Roslyn Francis

**Affiliations:** ^1^ The University of Western Australia, Western Australia National Imaging Facility, Perth, Western Australia, Australia; ^2^ The University of Western Australia, Perth, Western Australia, Australia; ^3^ Ear Science Institute Australia, Subiaco, Western Australia, Australia; ^4^ Sir Charles Gairdner Hospital, Nedlands, Western Australia, Australia; ^5^ Royal Perth Hospital, Perth, Australia; ^6^ University of Western Australia, Perth, Western Australia, Australia; ^7^ Harry Perkins Institute of Medical Research, The University of Western Australia, Perth, Western Australia, Australia

## Abstract

**Background:**

The HearCog trial was designed to evaluate the impact of hearing aid intervention on patients with age‐related hearing loss and cognitive impairment. An imaging substudy, utilising ^18^F‐FDG PET/CT as a functional imaging modality, was embedded to evaluate patterns of metabolic activity following early hearing aid intervention (group A) or delayed hearing aid intervention (group B).

**Method:**

28 study participants (mean age=77.4 years) underwent brain ^18^F‐FDG PET/CT imaging at baseline and 12 months, with *n* = 14 from each group. All imaging was undertaken using the same PET/CT camera (Biograph 64 mCT, Siemens Healthineers) accredited for quantitative assessment by the Australasian Radiopharmaceutical Trials Network. The ^18^F‐FDG dose was 185 MBq with a 10‐minute acquisition starting after 45 minutes uptake. For analysis of regional variation of ^18^F‐FDG brain metabolism, we employed the Molecular Imaging Neurology Database Comparison software application (Syngo.via MI Neurology DB Comparison, Siemens Healthineers) where ^18^F‐FDG brain scans from age‐matched (range 49‐79 years, *n* = 33) healthy volunteers were available for comparison. The analyses included semi‐automated anatomical co‐registration of subject images to the reference database, followed by an automated anatomical labelling of brain regions using the integrated atlas from Montreal Neurological Institute (MNI, McGill University). We compared the differences between the groups and the normal database for both timepoints, and performed parametric and non‐parametric statistical testing on differences between the study groups.

**Result:**

There were no significant differences between the groups in age, gender, injected ^18^F‐FDG activity, uptake time or days between imaging timepoints (*p* ≥0.06). Database analysis identified 5 regions associated with increased ^18^F‐FDG uptake between baseline and 12 months in the early (group A) compared to late (group B) intervention cohort. These regions include the central region (left and right), left central region, right rolandic operculum, left paracentral lobule and the left precentral gyrus.

**Conclusion:**

Analysis using standardised regions from Neurology Database comparison software demonstrates relative improvements in the motor/sensorimotor areas of the brain in the early compared to late intervention group. Further analysis will be undertaken integrating MRI and PET data for improved anatomical delineation in particular for evaluation of cognitive and auditory regions of the brain.

